# Hospital Meetings

**Published:** 1902-08-02

**Authors:** 


### Hospital Meetings.

### THE HOSPITAL FOR EPILEPSY AND PARALYSIS.

The thirty-fifth annual meeting of the Hospital for Epilepsy and Paralysis was held at the building of the new hospital, 4 Maida Vale, on July 23rd, when the Earl of Hardwicke was in the chair.

The secretary, Mr. H. Howgrave Graham, read the annual report, which contained a record of the most important event that had yet taken place in the annals of the hospital, namely, the bequest of the late Mr. A. T. Dettmar, which amounted to a net sum of £9,311. This handsome legacy had justified the committee in entering into a contract for the whole of the first section of the hospital, for a sum of £21,000. To completely fit and furnish this first section, however, the committee stand in urgent need of a sum of £2,500. As this hospital will, apparently, be the only one opened in 1902, the year of the King's Coronation, the committee considered that it seemed only fitting that it should start on its new career quite unhampered by debt.

The Earl of Hardwicke, in the course of his speech, referred briefly to the past history of the hospital and to the state of affairs which arose a few years ago when the committee found themselves faced with the necessity of either erecting a new hospital or abandoning the work of the charity altogether. They had decided to continue the work at all costs, and the generous bequest they had received had enabled them to practically carry out this decision. His lordship added that he was much pleased to see how fine a building it was, and in what a splendid situation it stood; it now only required the generous support of the public to accomplish most excellent work. The chairman then referred to the £2,500 that was needed to equip the hospital, and suggested that the best way of bringing the matter before the notice of the public would be to endeavour to secure the services of some prominent person for the opening ceremony. Lord Hardwicke concluded by a sympathetic reference to the death of Dr. Hughes Bennett, a consulting physician to the hospital.

Canon Duckworth, who was the next speaker, said that he took a special interest in the hospital, as it was situated in his own parish, and that he would do all in his power to promote the interests of this most useful hospital.

Major-General Sir Owen Tudor Burne offered some suggestions as to the alleged falling off of subscriptions to hospitals in London. One reason was that those who manage hospitals have usually more heart than head, and undertake extensions before the money has been provided and he hoped that the committee would take great care, after they had obtained the sum they now needed, not to launch out into further extensions before the funds were guaranteed. The new building being situated in the centre of a wealthy population, Sir Owen thought the clergy of the neighbourhood might be requested to make the work of the hospital and its needs known amongst their congregations. The speaker went on to say that he was glad to see that the patients were encouraged to contribute something to the funds of the hospital.

The architect of the new building, Mr. Keith Young, gave a slight description of the hospital, and stated that the whole building was built of fire-resisting materials, steel joists being used throughout. Accommodation was to be provided for the matron and six nurses.

A vote of thanks having been passed to the chairman the proceedings terminated.

